# Patterns of phenoloxidase activity in insecticide resistant and susceptible mosquitoes differ between laboratory-selected and wild-caught individuals

**DOI:** 10.1186/1756-3305-6-315

**Published:** 2013-10-31

**Authors:** Stéphane Cornet, Sylvain Gandon, Ana Rivero

**Affiliations:** 1 Maladies Infectieuses et Vecteurs: Ecologie, Génétique, Evolution et Contrôle (MIVEGEC), UMR CNRS 5290-IRD 224-UM1-UM2, Montpellier, France; 2 Centre d’Ecologie Fonctionnelle et Evolutive (CEFE), UMR CNRS 5175, Montpellier, France; 3 MIVEGEC, Institut de Recherche pour le Développement, 911 av. Agropolis, 34394, Montpellier, France

**Keywords:** *Culex*, Gender effect, Immune aging, Insecticide resistance, Phenoloxidase, Vector competence

## Abstract

**Background:**

Insecticide resistance has the potential to alter vector immune competence and consequently affect the transmission of diseases.

**Methods:**

Using both laboratory isogenic strains and field-caught *Culex pipiens* mosquitoes, we investigated the effects of insecticide resistance on an important component of the mosquito immune system: the phenoloxidase (PO) activity. As infection risk varies dramatically with the age and sex of mosquitoes, allocation to PO immunity was quantified across different stages of the mosquito life cycle.

**Results:**

Our results were consistent in showing that larvae have a higher PO activity than adults, females have a higher PO activity than males, and PO activity declines with adult age. We obtained, however, a marked discrepancy between laboratory and field-collected mosquitoes on the effect of insecticide resistance on PO activity. In the laboratory selected strains we found evidence of strong interactions between insecticide resistance and the age and sex of mosquitoes. In particular, 7 and 14 day old esterase-resistant adult females and acetylcholine-esterase resistant males had significantly higher PO activities than their susceptible counterparts. No such effects were, however, apparent in field-caught mosquitoes.

**Conclusions:**

Combined, the field and laboratory-based approaches employed in this study provide a powerful test of the effect of insecticide resistance on PO-mediated immunity. The use of laboratory-selected insecticide-resistant strains is still the most widely used method to investigate the pleiotropic effects of insecticide resistance. Our results suggest that the outcome of these laboratory-selected mosquitoes must be interpreted with caution and, whenever possible, compared with mosquitoes captured from the field.

## Background

Mosquito-borne diseases, such as malaria, dengue and filariasis represent a major cause of illness and death worldwide. The extensive use of chemicals to control mosquito populations and reduce the burden of disease has led to the widespread emergence and spread of insecticide resistance [1]. Insecticide resistance counters control methods by increasing vector population sizes above the critical thresholds required for disease management. Aside from its effect on vector numbers, however, insecticide resistance may also alter the quality of mosquitoes as vectors of disease [2,3]. In other words, insecticide resistant mosquitoes may be better (or worse) vectors of diseases than susceptible ones, a possibility which, if confirmed, could have drastic epidemiological and public health consequences [2].

There is a large body of work showing that the innate immune system is a key determinant of the vectorial capacity of mosquitoes: endogenous innate immune molecules hinder the development of malarial [4], filarial [5] and viral parasites [6]. In the last few years, a great deal of work has been undertaken to investigate the genetics and the molecular mechanisms underlying mosquito innate immune responses, on the one hand [7,8], and insecticide resistance, on the other [9]. In spite of this, few studies have investigated whether insecticide resistance and immune response are connected [10]. However, insecticide resistance genes, or genes closely associated to them as a result of hitchhiking (see e.g. [11,12]) could have a pleiotropic effect on one of the many steps of the immune cascade, from the recognition of the parasite as foreign, to the transduction of the signal and the deployment of the killing mechanism. For example, insecticide resistance may interfere with immunity through resource-based trade-offs, as both insecticide resistance mechanisms [13] and the deployment of the immune machinery [14,15] are energetically costly.

Here, we investigate whether insecticide resistance has a pleiotropic effect on the phenoloxidase activity of *Culex pipiens* mosquitoes. *Culex* mosquitoes are important vectors of diseases such as filariasis, West Nile, and Japanese encephalitis. *Cx. pipiens* has also a well-deserved reputation for being one of the mosquito species where the molecular and genetic bases of insecticide resistance are best understood [9,16]. The phenoloxidase cascade is an important immune reaction of mosquitoes leading to the melanotic encapsulation and death of a variety of parasites [17-21]. In *Cx. pipiens* it has been shown to be implicated in resistance against filarial parasites [22]. The melanotic encapsulation response results from the activation of a complex enzymatic cascade that ends up in the cleavage of inactive prophenoloxidases (proPO) into active phenoloxidases (PO), which initiate the production of melanin [23]. The activation of the cascade also generates toxic compounds that are efficient parasite killers [18,24]. The high diversification of the proPO genes revealed by comparative genomics has highlighted the importance of the melanization cascade in mosquito immunity [25,26]. In addition, the insect melanization response has been shown to be genetically correlated with other important components of the insect’s immune system [27,28], which has led to suggestions that the melanization cascade may be an indicator of the mosquito general immune competence.

The aim of the paper is to determine: (i) whether insecticide resistance affects mosquito PO activity and (ii) whether this effect varies between the larval and adult stages, and with the age and/or sex of the adults. We do the latter because allocation to immunity is a function of the level of parasite threat and of the costs associated to maintaining the trait [14], both of which vary widely throughout the life of a mosquito. *Cx. pipiens* larvae live in stagnant pools and are highly tolerant to organic and polluted water. As a result, larvae may be expected to have a stronger immune system than their adult counterparts (see e.g. [29]). Similarly, adult females may be expected to have a stronger immune system than males because blood feeding exposes them to a greater level and spectrum of parasites [30]. The immunity of both males and females is, however, expected to decline with age, as individuals shunt resources towards maximizing reproductive success before they die (immunosenescence, [31]). Immunosenescence is particularly relevant for disease vectors because, due to the long extrinsic incubation periods of many parasites, only relatively old individuals transmit diseases [32].

We carry out two separate experiments. In the first experiment (Experiment 1) we quantify PO activity in larvae, and in newly emerged (1-day), young (7-day) and old (14-day) male and female adults using three different isogenic *Cx. pipiens* strains: a fully susceptible strain, a strain resistant to insecticides through the overproduction of esterases (metabolic resistance), and a resistant strain with an insensitive acetylcholinesterase (target site resistance). The construction of isogenic laboratory strains (whereby the resistance alleles are introgressed into a susceptible reference line by a repeated backcross and insecticide selection procedure, e.g. [33]) remains one of the most widely used methods for investigating the pleiotropic effects of insecticide resistance in mosquitoes. This procedure has several advantages in that it allows testing the effect of the insecticide resistance genes in a uniform genetic background, which may increase the chances of detecting an eventual pleiotropic effect of the insecticide resistance genes. It also has, however, several potential pitfalls: (i) the selection procedure requires that these mosquitoes are subjected to unnaturally high insecticide selective pressures [34], (ii) the selection procedure may inadvertently select for other mosquito traits, and (iii) the results may not be necessarily applicable to other genetic backgrounds, particularly if there are epistatic interactions between the insecticide resistant genes and other genes in the background [10].

To account for these potential sources of bias, we therefore repeated the same experiment using field-caught mosquitoes (Experiment 2). In the Montpellier region, repeated treatments of *Cx. pipiens* larval sites with organophosphate insecticides (initiated 40 years ago) have resulted in the evolution of both esterase and acetylcholinesterase resistance. In this region, there is an insecticide-treated area (a 20 Km band close to the sea), a non-treated area (further north), and an intermediate area where esterase-resistant and acetylcholinesterase-resistant mosquitoes coexist with susceptible ones [35]. Combined, the field and laboratory-based approaches we employ in this study provide a powerful test of the role of insecticide resistance on PO activity throughout the mosquito life cycle.

## Methods

### Experiment 1 - Isogenic strain mosquitoes

We used three different isogenic strains of *Cx. pipiens* (SLAB, SA4B4 and SR) that share the same (SLAB) genetic background and only differ by their genotype at the *Ester* (esterase over-production) or the *ace-1* (insensitive acetylcholinesterase) loci: a fully susceptible strain (SLAB, alleles *Ester*^0^, *ace-1*^S^) and two strains resistant to insecticides, one through the overproduction of esterases A4 and B4 (SA4B4, alleles *Ester*^4^, *ace-1*^S^) and the other with an insensitive acetylcholinesterase but no overproduced esterases (SR, alleles *Ester*^0^, *ace-1*^R^) [16,33].

Synchronized *Cx. pipiens* L1 larvae cohorts from the three insecticide resistant strains were obtained from the Weill’s lab at the Institut des Sciences de l’Evolution de Montpellier (France) and maintained under our standard insectary conditions (12 h:12 h photoperiod, 25 ± 1°C, 75 ± 5% RH). Larvae were reared in plastic trays (25 × 35 × 7 cm, 4 trays per strain) filled with 1 L of source water (Carrefour) at an initial density of 300. Larvae were fed with fish food (Microfood, Tretramin®) and water was renewed each time food was provided. Each tray was placed inside a mesh cage (27 × 40 × 35 cm) for adult emergence (around day 10). Adults were provided with an *ad libitum* 10% sugar solution for the duration of the experiment.

### Experiment 2- Field-caught mosquitoes

Wild *Cx. pipiens* mosquito eggs were collected in June-July 2011 from a population (Triadou, 20 km north Montpellier, France) where the insecticide susceptible (*Ester*^0^, *ace-1*^S^) and resistant (*Ester*^4^, *ace-1*^R^) alleles are found in sympatry [36]. After hatching, 4 trays of 400 L1 larvae each were set up. Mosquitoes were reared and maintained under identical laboratory conditions as described above.

### Experimental procedure

Both experiments were performed in an identical manner. The immune tests were carried out at four different stages of the mosquito life cycle: L4 larvae (7 days post-hatching), newly emerged adults, young adults and old adults (1, 7 and 14 days post-emergence, respectively). In Experiment 1, at each time step, either 20 larvae or 30 adults (15 males and 15 females) per tray (or, in the case of adults, cage) were haphazardly sampled from the pool of individuals. In Experiment 2 we sampled either 40 larvae or 80 adults (40 males and 40 females). The higher replication number was to account for the fact that this experiment had to be done blind: assignation to a particular insecticide resistance or susceptible genotype was done *a posteriori* (see “Mosquito genotyping” below)*.* Since females emerged on average one day later than males, we respected a one-day shift in the sampling of males and females. Mosquito size was recorded by measuring (i) the thorax width of larvae and (ii) wing size of adults, using a binocular microscope linked to an image analysis software.

### Measures of phenoloxidase activity

Melanization results from the enzymatic activation of prophenoloxidases (proPO) into phenoloxidases (PO), which initiate the production of melanin. Both the ready-to use PO (henceforth “active PO”) and its proPO precursor are stored in the haemolymph. While the quantification of the active PO can be assessed directly (see below), the estimation of the overall investment in the PO pathway (henceforth “total PO”) requires the prior transformation of proPO into PO using chymotrypsin. Biologically speaking, “active PO” measures the amount of PO naturally activated, while “total PO” measures the overall investment in this immune pathway (i.e. active PO plus inactive proPO, see also [37]).

As the amount of haemolymph that can be extracted from a single *Culex* mosquito is very limited, phenoloxidase activity was quantified using individual homogenates. As there is increasing evidence that in mosquitoes haemocytes in the haemolymph are the main source of proPO/PO synthesis [38,39], this is unlikely to have biased our results. For this purpose, mosquitoes (larvae and adults) were crushed individually in 35 μL of cold PBS buffer. After centrifugation (2 min, 4°C, 3000 rpm), 15 μL of the supernatant was collected, immediately frozen in liquid nitrogen and stored at -80°C for subsequent analysis.

The analyses of the active PO and total PO activities were carried out using a spectrophotometric assay [37]. The assay was performed using 5 μL of supernatant extract added to a microplate well containing 20 μL of PBS buffer and either 140 μL of dH_2_O to measure active PO, or 140 μL of chymotrypsin solution (Sigma C-7762, 0.07 mg/mL of dH_2_O) to measure total PO activity. In both cases, 20 μL of L-Dopa solution (the substrate for the PO, Sigma D-9628, 4 mg/mL of dH_2_O) was added to the mixture and the colorimetric reaction was followed in a microplate reader (Versamax, Molecular Devices) at 30°C. Readings were taken for 30 min at 490 nm. Enzyme activity was analysed using the software SOFT-Max®Pro 5.2 (Molecular Devices) and measured as the slope (V_max_ value) of the reaction curve during the linear phase. The values reported refer to the activity present in a standard 1 μL volume of supernatant. In experiment 1, total and active PO were highly correlated and provided nearly identical results (see below). Due to a technical problem (faulty chymotrypsin), however, we were unable to reliably quantify total PO activity in experiment 2.

### Mosquito genotyping

Field-caught mosquitoes (experiment 2) need to be typed to determine their insecticide resistance status. Once the haemolymph was collected and analysed for PO activity (see above), the mosquito was homogenised and the genomic DNA was extracted using the procedure outlined in the DNeasy Blood & Tissue Kit (Qiagen). Insecticide resistance through the overproduction of carboxylesterases was analysed using RFLP analysis at the *Ester-3* gene [40]. This technique allows us to distinguish between susceptible mosquitoes (allele *Ester*^*0*^), and the most common insecticide resistant carboxylesterase allozyme present in the study area: A4-B4 (allele *Ester*^*4*^). Insecticide resistance through the modification of the acetylcholinesterase was established using RFLP analysis at the *ace-1* gene [41]. This technique allows us to distinguish between susceptible (allele ace-1^*s*^) and target-site resistant (allele ace-1^*s*^) mosquitoes.

Overall, 824 individuals were successfully allocated to one of three groups depending on their insecticide resistant status (Additional file 1: Table S1): S (fully susceptible, *Ester*^*0*^*,* ace-1^*s*^), E (resistant through esterase overproduction, *Ester*^*4*^*,* ace-1^*s*^), and R (resistant through acetylcholinesterase modification, *Ester*^*0*^*,* ace-1^*R*^). We failed to obtain a reliable genotype for 165 samples, and 130 other samples were discarded because of mixed resistance or rare *Ester-3* genotypes (A1, A2 or a mixed-allele combination).

### Statistical analyses

The statistical analyses were run using the R software (v. 2.14.0). Variation in phenoloxidase activity was analyzed using linear mixed-effect models (*lme* function, *nlme* package). Tray (larvae) or cage (adults) effects were included as random factors, and insecticide resistance (IR), stage (larval/adult) age (adults only), sex (adults only) and size as fixed factors. Models were simplified by sequentially eliminating the least non-significant term to obtain minimal adequate models using a standard procedure of likelihood comparison (using the function anova.lme and specifying a marginal type test). When appropriate, *a posteriori* contrasts were carried out by aggregating factor levels that did not significantly differ from each other and by testing the fit of the simplified model [42].

## Results

### Experiment 1- Isogenic strain mosquitoes

**Figure 1:**
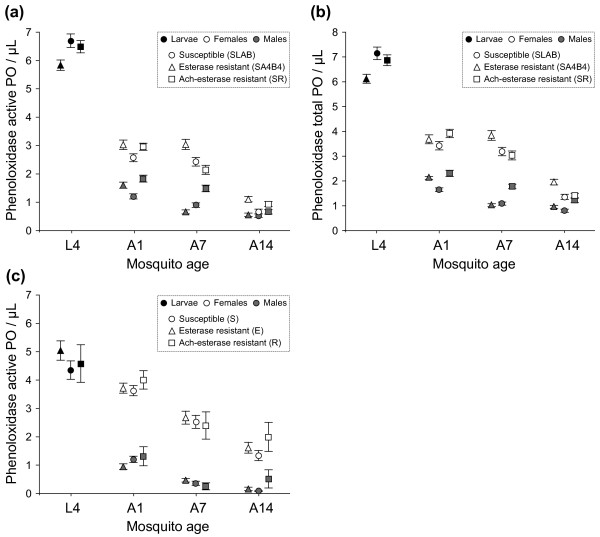
**Levels of phenoloxidase activity (active PO and total PO) per microlitre of homogenate sample (mean ± se) in unsexed L4 larvae (dark symbols), and in male (grey symbols) and female (white symbols) adults of *Culex pipiens *mosquitoes.** Adults were either 1-day old (A1), 7-days old (A7) or 14-days old (A14). Experiment 1: **(a)** active PO and **(b)** total PO in isogenic strain mosquitoes: insecticide-susceptible mosquitoes (SLAB strain, circles), esterase resistant mosquitoes (SA4B4 strain, triangles), acetylcholinesterase resistant mosquitoes (SR strain, squares). Experiment 2: **(c)** active PO in field-caught mosquitoes: insecticide-susceptible mosquitoes (S, circles), esterase resistant mosquitoes (E, triangles), acetylcholinesterase resistant mosquitoes (R, squares).

The total and active PO activities per μL homogenate were highly correlated (*r* = 0.97, *t*_1, 1313_ = 155.58, *P* < 0.0001). Insecticide resistance affects both the active and total PO activities (active PO: *F*_2, 232_ = 4.89, *P* = 0.0083; total PO: *F*_2, 232_ = 6.70, *P* = 0.0006) of mosquito larvae. Esterase resistant (SA4B4) larvae have significantly less active and total PO activities per μL than either susceptible (SLAB) or acetylcholinesterase resistant (SR) larvae (Figure 1a, b; contrast analysis SA4 *vs* SLAB + SR, active PO: χ^2^_1_ = 8.68, *P* = 0.0032; total PO: χ^2^_1_ = 13.11, *P* = 0.0003). Larval size is positively related to both active PO (*F*_1, 232_ = 94.73, *P* < 0.0001) and total PO (*F*_1, 232_ = 111.80, *P* < 0.0001), but this effect is independent of the insecticide resistance strain (IR*size interaction, active PO: *F*_2, 230_ = 0.84, *P* = 0.4342; total PO: *F*_2, 230_ = 0.57, *P* = 0.5665; the size of L4 larvae does not differ between strains, *F*_2, 233_ = 0.35, *P* = 0.7055).

Adult emergence is characterized by an intense reduction of the phenoloxidase activity (Figure 1a, b). Larvae (mean ± se, active PO: 6.34 ± 0.12, total PO: 6.71 ± 0.12) have roughly more than twice the amount of active PO (2.20 ± 0.06; *stage* effect *F*_1, 590_ = 1037.46, *P* < 0.0001) and total PO (2.85 ± 0.07; *F*_1, 590_ = 776.16, *P* < 0.0001) per μl homogenate than newly emerged adults (analysis carried out pooling together newly emerged males and females, see Figure 1a, b).

The full model containing all explanatory factors rendered a very significant three way IR*age*sex interaction for adult active and total PO activities (*F*_4, 1055_ = 4.87, *P* = 0.0007 and *F*_4, 1055_ = 4.96, *P* = 0.0006, respectively; Additional file 1: Table S2). In order to investigate this three-way interaction in more detail we decomposed it into two separate analyses (Tables 1 and 2).

**Table 1:** **Variation in phenoloxidase activity of adult *Culex pipiens *** **mosquitoes according to age, sex and size**

	**(a) Isogenic mosquitoes**				**(b) Wild mosquitoes**	
	**Active PO**		**Total PO**		**Active PO**	
**Factor**	***F***_**(df)**_	***P***	***F***_**(df)**_	***P***	***F***_**(df)**_	***P***
Age	247.17 _(2, 1065)_	< 0.0001	214.62 _(2, 1065)_	< 0.0001	111.15 _(2, 734)_	< 0.0001
Sex	29.43 _(1, 1065)_	< 0.0001	15.10 _(1, 1065)_	0.0001	1.24 _(1, 734)_	0.2661
Size	4.87 _(1, 1065)_	0.0275	0.31 _(1, 1065)_	0.5745	20.71 _(1, 734)_	< 0.0001
Age*sex	58.25 _(2, 1065)_	< 0.0001	51.78 _(2, 1065)_	< 0.0001	19.66 _(2, 734)_	< 0.0001
Sex*size	22.33 _(1, 1065)_	< 0.0001	10.39 _(1, 1065)_	0.0013	3.84 _(1, 734)_	0.0504

(a) Experiment 1 using isogenic strain mosquitoes. (b) Experiment 2 using wild mosquitoes.

**Table 2:** **Variation in phenoloxidase activity of adult female and male *Culex pipiens *** **mosquitoes according to insecticide resistance genotype (IR), age and size**

	**(a) Isogenic mosquitoes**				**(b) Wild mosquitoes**	
	**Active PO**		**Total PO**		**Active PO**	
**Factor**	***F***_**(df)**_	***P***	***F***_**(df)**_	***P***	***F***_**(df)**_	***P***
*Females*						
IR	3.22 _(2, 525)_	0.0409	2.62 _(2, 525)_	0.0735	0.27 _(2, 348)_	0.7658
Age	67.99 _(2, 525)_	< 0.0001	47.62 _(2, 525)_	< 0.0001	25.79 _(2, 348)_	< 0.0001
Size	0.02 _(1, 525)_	0.8845	4.19 _(1, 525)_	0.0412	12.13 _(1, 348)_	0.0006
IR*age	2.66 _(4, 525)_	0.0319	3.39 _(4, 525)_	0.0094	0.45 _(4, 348)_	0.7756
*Males*						
IR	10.71 _(2, 525)_	< 0.0001	10.29 _(2, 525)_	< 0.0001	3.22 _(2, 371)_	0.0409
Age	40.31 _(2, 525)_	< 0.0001	42.32 _(2, 525)_	< 0.0001	24.71 _(2, 371)_	< 0.0001
Size	12.64 _(1, 525)_	0.0004	19.85 _(1, 525)_	< 0.0001	5.25 _(1, 371)_	0.0225
IR*age	4.07 _(4, 525)_	0.0029	2.90 _(4, 525)_	0.0215	2.41 _(4, 371)_	0.0490

(a) Experiment 1 using isogenic strain mosquitoes. (b) Experiment 2 using wild mosquitoes.

In the first analysis, we pooled all IR strains together and we investigated the effect of mosquito age and sex on PO activity. This analysis shows that females have significantly higher active and total PO activities per μL homogenate than males (Table 1a). The difference between the sexes is particularly striking on days 1 and 7, but tends to decrease later in life (day 14, Figure 1a, b). In both males and females, PO decreases as the mosquitoes age, but the pattern of reduction is markedly different between the sexes (significant age*sex interaction, Table 1a). Males show a monotonic decrease in PO activity as they age. In contrast, female active and total PO activities are maintained until day 7 before decreasing drastically on day 14.

In the second analysis we explored the effect of insecticide resistance and age on PO activity separately for males and females (Table 2a). In both sexes, the pattern of PO decay is markedly different between the insecticide-resistant and susceptible strains (significant IR*age interaction, Table 2a). In 1-day old adult females, there was a trend towards both of the insecticide resistant strains (SA4 and SR) having higher PO levels than their susceptible (SLAB) counterparts, although this trend was only significant for the active PO (active PO: χ^2^_1_ = 5.74, *P* = 0.0166; total PO: χ^2^_1_ = 3.95, *P* = 0.1386). In 7 and 14-day old females, however, it is only the esterase resistant (SA4B4) strain which has significantly higher levels of both total and active PO activity than SLAB and SR females (contrast analyses; active PO: day 7: χ^2^_1_ = 13.98, *P* = 0.0002; day 14: χ^2^_1_ = 248.84, *P* < 0.0001; total PO: day 7: χ^2^_1_ = 10.90, *P* = 0.0010; day 14: χ^2^_1_ = 215.82, *P* < 0.0001; Figure 1a, b). In addition, whereas both SLAB and SA4B4 females maintained their level of PO activity for the first 7 days, SR females lost about 30% of their PO activity in the same period (Figure 1a, b). In 1-day old males the pattern was similar to that of females: resistant (SA4B4 and SR) strains had significantly higher PO activity levels than their susceptible (SLAB) counterparts (active PO: χ^2^_1_ = 12.64, *P* < 0.0001; total PO: χ^2^_1_ = 14.17, *P* < 0.0001). However, in 7 and 14-day old males it is the acetylcholinesterase resistant (SR) strain which shows significantly higher PO levels than the other two strains (contrast analyses; active PO: day 7: χ^2^_1_ = 35.83, *P* = 0.0002; day 14: χ^2^_1_ = 131.41, *P* < 0.0001; total PO: day 7: χ^2^_1_ = 35.01, *P* < 0.0001; day 14: χ^2^_1_ = 135.25, *P* < 0.0001; Figure 1a, b). Whereas PO activity in SLAB and SR males decreased linearly with time, it was halved in SA4 males between day 1 and day 7.

### Experiment 2- Field-caught mosquitoes

Due to a technical problem (see above), in experiment 2, only active PO measurements could be obtained. The active PO of field-caught larvae is identical for the three insecticide resistance genotypes (*F*_2, 72_ = 1.05, *P* = 0.3565; Figure 1c). Larval size is positively correlated with active PO (*F*_1, 72_ = 65.08, *P* < 0.0001), but this relationship is not significantly affected by the insecticide resistance genotype (IR*size interaction, *F*_1, 70_ = 2.67, *P* =0.0763; the size of L4 larvae does not differ between strains, *F*_2, 73_ = 1.34, *P* = 0.2690).

As was the case for the isogenic strain mosquitoes, the transition from larvae to newly emerged adult is characterized by a large (2-fold) reduction in the amount of active PO per μL homogenate (larvae 4.77 ± 0.22, adults A1 2.41 ± 0.11; *stage* effect *F*_1, 315_ = 108.02, *P* < 0.0001; analysis was carried out pooling together newly emerged males and females, see Figure 1c).

Also congruent with the isogenic strain experiments is the fact that females have significantly higher levels of active PO per μL homogenate than males (Table 1b). In both males and females, PO decreases significantly as the mosquitoes age, although the pattern of reduction differs between the sexes (significant age*sex interaction, Table 1b). The decreased PO activity with age was stronger for females than for males (Figure 1c).

We explored the effect of insecticide resistance and adult age on PO immune activity separately for males and females (Table 2b). In both sexes, active PO decreases significantly with age (Table 2b). The most striking difference with the isogenic strain experiments is that here insecticide resistance does not have any noticeable effect on PO immune activity (Figure 1c).

## Discussion

### Insecticide resistance

In insects, the evolution of insecticide resistance has often been found to be associated to life history costs (see however [43]). These costs are largely expected to appear in mosquitoes that are resistant to insecticides through the overproduction of detoxifying enzymes, because of the large amount of resources that need to be diverted for the large-scale production of the extra proteins [2]. Consistent with this, we and others have shown that *Cx. pipiens* females resistant to insecticides through the overproduction of esterases have 30% less energetic resources [13], a higher pre-imaginal mortality [44] and a significantly shorter lifespan [45,46] than susceptible ones. A reasonable prediction is therefore that these mosquitoes may also be less immunocompetent than their susceptible counterparts, which if true, could have dramatic consequences for the transmission of diseases. The PO activity of our field-caught esterase-resistant and acetylcholinesterase-resistant females was, however, equal to that of susceptible females. More surprisingly, the results of the isogenic laboratory strains showed that insecticide resistant mosquitoes tend to have higher, as opposed to lower PO activities. In particular, young (7-day) and, to a lesser extent, old (14-day) esterase-resistant (SA4B4) adult females had a significantly higher PO activity than individuals of the other two strains. The same is true for young and old acetylcholinestrase-resistant males, although the reasons for the differences between the sexes are not entirely clear. Intriguingly, these unexpected results are in agreement with several other studies comparing the immunity of insecticide resistant and susceptible mosquito populations using laboratory-selected strains. One straightforward explanation is that the strong insecticide doses used to select for resistance in the laboratory selects for insects with higher PO levels because PO plays a key role in cuticle sclerotization, and as the cuticle is a major route for insecticide penetration, insecticide resistance is often associated to thicker/harder cuticles [47,48]. Contrary to this hypothesis is the fact that no such increased PO activity was observed in acetylcholinesterase-resistant females nor in esterase-resistant males, despite them being subject to similarly high insecticide pressures. Furthermore, the increased immune activity of insecticide resistant insects is not restricted to PO. Using the same *Cx. pipiens* laboratory strains, we recently quantified the antibacterial peptide and nitric oxide synthase (NOS) transcripts of 7-day old insecticide resistant and susceptible *Cx. pipiens* females either before (constitutive) or after (induced) an LPS challenge. Insecticide resistant isogenic strains were shown to have a significantly enhanced expression of virtually all the immune related genes investigated [10]. These results agreed with previous microarray studies carried out in two other species: *Anopheles gambiae*[49] and *An. stephensi*[47], where it was also shown that several antibacterial peptides and the NOS gene were constitutively transcribed at a higher level in laboratory-maintained insecticide-resistant strains of mosquitoes than in their insecticide susceptible counterparts. One pervasive criticism addressed at mRNA quantification studies is, however, that they do not necessarily reflect the level of immunity of the organism, as regulation can take place downstream from the transcription step [50]. The results of the present study show that the pleiotropic effects of insecticide resistance on the immunity of laboratory-selected mosquitoes are also seen at the level of an immune effector (PO).

The mechanisms underlying the higher PO activity in esterase-overproducing laboratory females are not clear, but at least three potential scenarios are possible. Esterase overproduction in *Cx. pipiens* is affected through the co-amplification of two paralogous esterase loci: *Est-3* (encoding for the esterase A) and *Est-2* (esterase B). The amplicons on which these esterases occur are, however, much larger (30-60 kb) than the esterase containing region (~10 kb, [11]). So far, only one other gene present in the amplicon of insecticide resistant *Cx. pipiens* has been identified (aldehyde oxydase, [11]) but there could be others. One possibility is that one of the immune genes implicated in the complex enzymatic cascade that leads to the activation of PO [23] or one of their *trans*-acting regulatory genes, is coamplified with the esterases. Alternatively, the original esterase resistance allele used to create the SA4B4 strain [33,51] may have been in linkage disequilibrium with genes involved in the melanisation cascade. Such associations have been found before. For example, in a particular strain of *An. gambiae*, the genes responsible for *Plasmodium* melanization seem to be in the same chromosomal inversion as an esterase locus that encodes for a protein very similar to the insecticide-conferring carboxylase from *Cx. pipiens*[52].

Finally, it is not impossible that the strong immune phenotype observed in the isogenic SA4B4 strain may be the result of epistatic interactions between the insecticide resistance genes (or genes closely associated with them at the amplicon or allelic level), and the SLAB genetic background. The finding, however, that selection for high resistance levels in laboratory strains from two other mosquito species also results in an upregulation of the immune system [47,49] suggests that our results are not specific to a particular genetic background and that the effect may be a common artefact of laboratory strains.

Irrespective of the underlying mechanism, our results add weight to claims that the results of experiments using laboratory-selected insecticide-resistant strains, still the most widely used method to investigate the pleiotropic effects of insecticide resistance, must be interpreted with caution [2,34,53] and, whenever possible, experiments should be done using wild-caught mosquitoes. Unfortunately, due to the long and complex history of insecticide use in most mosquito-infected areas, which result in most individuals being resistant at, at least, one of the many possible insecticide resistant loci, the opportunities for comparing field-caught sympatric insecticide resistant and susceptible mosquitoes are few and far between, so the options for carrying meaningful comparative studies are limited. On the other hand, the greater genetic and environmental variances and the constraints on sample sizes that are inevitably associated with wild-caught mosquitoes (apparent in our experiment by smaller sample sizes and larger error bars in our field-caught acetylcholinesterase-resistant mosquitoes) may reduce the statistical power to detect a difference.

### Mosquito stage, sex and age

Laboratory and field-caught mosquitoes were consistent in showing that: (i) larvae have a higher PO activity than adults, (ii) adult females have a higher PO activity than males and (iii) PO activity declines with adult age.

The process of metamorphosis that takes place in holometabolous insects has often been considered to be an adaptive strategy to decouple the life history traits of larvae and adults [54]. Recent work, however, shows that, at least for the immune system, there is a certain degree of continuity between the larval and adult stages. For example, Fellous and Lazzaro [55] have shown a great degree of genetic correlation in the transcription of certain antibacterial peptides between the larval and adult stages of *Drosophila melanogaster.* Other studies have shown that the larval conditions determine the immunity and vectorial capacity of the adults [56-58]. Despite this, we know surprisingly little about larval immunity in mosquitoes (but see [59]). One general prediction is that larvae should have a stronger immune system than adults because their aquatic life style exposes them to a higher density and diversity of parasites. *Cx. pipiens* larvae can be found in a fairly wide range of larval habitats but are generally associated with water that has a high organic and bacterial content (such as sewage treatment plants). The high PO activity we found in both the laboratory and the field-caught larvae may be consistent with this hypothesis. Interestingly, in the isogenic laboratory strains, the PO activity per μL homogenate was twice as high in larvae as in adults (Figure 1a, b). The effect was, however, less drastic in field-caught mosquitoes, particularly in females (Figure 1c), a result that may be explained by the fact that wild females are exposed to a larger density and diversity of blood-associated parasites than laboratory-reared ones. Given that PO activities were assessed from crushed extracts and that we could not have a common size estimator for larvae and adults (i.e. thorax width and wing size, respectively), the comparison of immune activity between mosquito stages needs to be taken with caution.

The difference in immune investment we found between males and females agrees with previous data from other invertebrates [60,61]. This can be explained by two non-exclusive factors. First, by differences in lifespan and in the constraints associated with their respective reproductive schedules [62,63]. Indeed, males of most species, including mosquitoes, have what Zuk [64] described as a “live hard, die young” strategy. In contrast, the female reproductive life is made up of lengthy gonotrophic cycles, each of them consisting of a period of host seeking, followed by blood feeding, egg maturation and oviposition which, collectively, can last 2-5 days depending on the species [65]. For females, therefore, longevity is an essential component of their reproductive fitness and surviving parasites is, by extension, potentially much more important. Secondly, by sex-associated differences in parasite exposure. While *Cx. pipiens* males feed exclusively on nectar [65], which plants keep mostly bacterial and fungi-free [66], females are exposed to a wide array of blood-associated pathogens that include viruses (West Nile and encephalitis), protozoan (*Plasmodium*, *Trypanosoma*) and metazoan (*Wuchereria*) parasites [3,67,68]. To verify that these *a posteriori* explanations for our results hold, an interesting experiment would, therefore, be to compare the immune competence in males and females of species, such as the tsetse fly (*Glossina sp.*), where both sexes blood feed and have similar lifespans [69]. Despite using whole body extracts to quantify the PO activity, the difference between males and females found here are unlikely to have been biased by the presence of ovaries in the mixture. PO does indeed play a key role in egg production, but this role seems to be restricted to the tanning of the chorion, which is secreted just before the eggs are laid [39]. Previous reports of PO activity in other tissues such as ovaries may, in fact, be due to contamination with haemocytes [38,39]. In mosquitoes, PO in the ovaries is therefore largely associated to the presence of mature (chorionated) eggs. However, since in our experiment mosquitoes were not allowed to blood feed, they did not contain any mature eggs in the ovarioles.

Although the connection between age and immunocompetence has been well established in insects, its occurrence in mosquitoes has been largely ignored or given only cursory attention [70,71], and this despite earlier reports showing that parasite infection intensity is higher older mosquitoes [72,73]. Our results show that, in female mosquitoes in particular, PO activity declines markedly with age, so that by day 14 females have lost between two thirds (isogenic mosquitoes) and a half (field mosquitoes) of their PO at emergence (Figure 1a, b and c). Such immune senescence is less marked in males possibly because of the lower PO levels at emergence that does not leave much room for further reductions. Aging has been previously associated with a decrease in PO activity and/or a decreased ability to encapsulate parasites in several insect species [71,73-79]. The decrease in PO with age may not, however, necessarily indicate a generalized immune senescence but be a result of a plastic adjustment of immune function with age. Moret and Schmid-Hempel [80], for example, found that in bumble bees PO and antimicrobial activity strongly trade-off with each other: as colonies age the decrease in PO activity is associated with an increase in antimicrobial activity, even in the presence of plentiful resources. In mosquitoes, however, no such age-associated change has been detected for antimicrobial peptide production [71]. In addition, the evidence available suggests that in mosquitoes the melanisation and antibacterial responses are positively correlated [28].

Immune decay has been described in many invertebrate species, but its consequences are particularly poignant for mosquitoes because only relatively old females transmit diseases. This correlation between mosquito age and parasite transmission is due to the combined effects of (1) a long time lag between adult emergence and the activation of the host seeking behaviour (between 2-5 days for most species, [65]), (2) the interval between blood feeding and laying events imposed by the gonotrophic cycles (2-4 days, [65]), and (3) the long extrinsic incubation period of most mosquito-transmitted parasites (10-14 days for filaria, West Nile and encephalitis viruses, [81-83]).

## Conclusions

Mosquito immunocompetence cannot be reduced to a single immunological measurement [84,85]. However, the PO cascade is a key component of insect host condition, hemostasis and immunity and is undoubtedly a major player in the fight against a wide range of parasites [17]. We found no effect of insecticide resistance on the PO activity of field-caught *Cx. pipiens* mosquitoes. Further work is needed to determine whether these results extend to other mosquito species, immune effectors and insecticide resistant mechanisms, and to examine the consequences of insecticide resistance on vector competence to pathogens [36,86]. We also observed a significant decline in PO activity with mosquito age. Despite its potential consequences for parasite transmission, immune senescence in mosquitoes has been little studied (but see [71,73,76]). Assuming that PO measurements correlate with disease resistance, our results predict that, everything else being equal, older mosquitoes may show a higher prevalence and/or intensity of infection than younger, more immunocompetent ones. We are, however, not aware of any studies that have compared the vectorial capacity of mosquitoes of different ages and further work is needed to demonstrate an association between levels of PO activity and resistance to parasitism in mosquitoes (especially wild-caught mosquitoes). In addition, an interesting spin-off of these results is that late life acting (or “evolution proof”) insecticides, whose purpose is to kill mosquitoes after they have reproduced but before they are able to transmit parasites [87,88] would have the additional advantage of killing the least immune competent (and thus, potentially, most transmission-efficient) fraction of the mosquito population.

## Competing interests

The authors declare that they have no competing interests.

## Authors’ contributions

SC, SG and AR conceived and designed the experiment. SC performed the experiment and analysed the data. SC, SG and AR wrote the paper. All authors read and approved the final version of the manuscript.

## Supplementary Material

Additional file 1 ***Supplementary materials. *Table S1**: Mosquito sample size of the different insecticide resistance groups (insecticide-susceptible mosquitoes (SLAB/S), esterase resistant mosquitoes (SA4B4/E), acetylcholinesterase resistant mosquitoes (SR/R) according to stage (larvae, adults), age (1, 7, 14 days) and sex (males, females). (a) Experiment 1 using isogenic strain mosquitoes. (b) Experiment 2 using wild mosquitoes. **Table S2**: Variation in phenoloxidase activity of adult *Culex pipiens* mosquitoes according to insecticide resistance (IR), age and sex. (a) Experiment 1 using isogenic strain mosquitoes. (b) Experiment 2 using wild mosquitoes. Click here for file
