# Supplementary material for: Patterns of phenoloxidase activity in insecticide resistant and susceptible mosquitoes differ between laboratory-selected and wild-caught individuals
Source: Parasit Vectors. 2013 Oct 31;6:315. doi: 10.1186/1756-3305-6-315 (PMC3819646; doi:10.1186/1756-3305-6-315)
Supplement: Additional file 1 — Supplementary materials. Table S1: Mosquito sample size of the different insecticide resistance groups (insecticide-susceptible mosquitoes (SLAB/S), esterase resistant mosquitoes (SA4B4/E), acetylcholinesterase resistant mosquitoes (SR/R) according to stage (larvae, adults), age (1, 7, 14 days) and sex (males, females). (a) Experiment 1 using isogenic strain mosquitoes. (b) Experiment 2 using wild mosquitoes. Table S2: Variation in phenoloxidase activity of adult Culex pipiens mosquitoes according to insecticide resistance (IR), age and sex. (a) Experiment 1 using isogenic strain mosquitoes. (b) Experiment 2 using wild mosquitoes. [file 1756-3305-6-315-S1.doc]

**SUPPLEMENTARY MATERIALS**

**Table S1.** Mosquito sample size of the different insecticide resistance groups (insecticide-susceptible mosquitoes (SLAB/S), esterase resistant mosquitoes (SA4B4/E), acetylcholinesterase resistant mosquitoes (SR/R) according to stage (larvae, adults), age (1, 7, 14 days) and sex (males, females). (a) Experiment 1 using isogenic strain mosquitoes: . (b) Experiment 2 using wild mosquitoes.

|  |  |  | (a) Isogenic mosquitoes | | | (b) Wild mosquitoes | | |
| --- | --- | --- | --- | --- | --- | --- | --- | --- |
|  |  |  | SLAB | SA4 | SR | S | E | R |
| Larvae | L4 |  | 79 | 80 | 80 | 31 | 39 | 9 |
| Adults | A1 | Males | 58 | 60 | 60 | 52 | 64 | 11 |
|  |  | Females | 60 | 59 | 59 | 52 | 61 | 10 |
|  | A7 | Males | 60 | 60 | 60 | 51 | 69 | 8 |
|  |  | Females | 60 | 60 | 60 | 55 | 54 | 12 |
|  | A14 | Males | 60 | 60 | 60 | 52 | 63 | 14 |
|  |  | Females | 60 | 60 | 60 | 62 | 43 | 12 |

**Table S2.** Variation in phenoloxidase activity of adult *Culex pipiens* mosquitoes according to insecticide resistance (IR), age and sex. (a) Experiment 1 using isogenic strain mosquitoes. (b) Experiment 2 using wild mosquitoes.

|  | (a) Isogenic mosquitoes | | | | (b) Wild mosquitoes | |
| --- | --- | --- | --- | --- | --- | --- |
|  | Active PO | | Total PO | | Active PO | |
| Factor | *F* _(df)_ | *P* | *F* _(df)_ | *P* | *F* _(df)_ | *P* |
| IR | 4.54 _(2, 1055)_ | 0.0190 | 3.27 _(2, 1055)_ | 0.0385 | 0.49 _(2, 724)_ | 0.6153 |
| Age | 92.93 _(2, 1055)_ | < 0.0001 | 64.40 _(2, 1055)_ | < 0.0001 | 44.88 _(2, 724)_ | < 0.0001 |
| Sex | 76.17 _(1, 1055)_ | < 0.0001 | 70.52 _(1, 1055)_ | < 0.0001 | 190.87 _(1, 724)_ | < 0.0001 |
| IR*age | 3.67 _(4, 1055)_ | 0.0056 | 4.38 _(4, 1055)_ | 0.0016 | 0.71 _(4, 724)_ | 0.5861 |
| IR*sex | 0.95 _(2, 1055)_ | 0.3872 | 0.46 _(2, 1055)_ | 0.6293 | 0.68 _(2, 724)_ | 0.5047 |
| Age*sex | 31.46 _(2, 1055)_ | < 0.0001 | 25.70 _(2, 1055)_ | < 0.0001 | 9.72 _(2, 724)_ | 0.0001 |
| IR*age *sex | 4.87 _(4, 1055)_ | 0.0007 | 4.96 _(4, 1055)_ | 0.0006 | 0.15 _(4, 724)_ | 0.9647 |
